# A Diploblastic Radiate Animal at the Dawn of Cambrian Diversification with a Simple Body Plan: Distinct from Cnidaria?

**DOI:** 10.1371/journal.pone.0065890

**Published:** 2013-06-20

**Authors:** Kinya Yasui, James D. Reimer, Yunhuan Liu, Xiaoyong Yao, Daisuke Kubo, Degan Shu, Yong Li

**Affiliations:** 1 Department of Biological Sciences, Graduate School of Science, Hiroshima University, Higashi-hiroshima, Hiroshima, Japan; 2 Rising Star Program, Trans-disciplinary Organization for Subtropical Island Studies, University of the Ryukyus, Nishihara, Okinawa, Japan; 3 University Museum of Geology, Chang'an University, Xi'an, People's Republic of China; 4 State Key Laboratory of Continental Dynamics, Department of Geology, Early Life Institute, Northwest University, Xi'an, Shaanxi, People's Republic of China; 5 Department of Biological Sciences, Graduate School of Science, The University of Tokyo, Bunkyo-ku, Tokyo, Japan; 6 School of Earth Science and Land Resources, Key Laboratory of Western China's Mineral Resources and Geological Engineering, Chang'an University, Xi'an, People's Republic of China; UC Irvine, United States of America

## Abstract

**Background:**

Microfossils of the genus *Punctatus* include developmental stages such as blastula, gastrula, and hatchlings, and represent the most complete developmental sequence of animals available from the earliest Cambrian. Despite the extremely well-preserved specimens, the evolutionary position of *Punctatus* has relied only on their conical remains and they have been tentatively assigned to cnidarians. We present a new interpretation of the *Punctatus* body plan based on the developmental reconstruction aided by recent advances in developmental biology.

**Results:**

*Punctatus* developed from a rather large egg, gastrulated in a mode of invagination from a coeloblastura, and then formed a mouth directly from the blastopore. Spiny benthic hatchlings were distinguishable from swimming or crawling ciliate larvae found in cnidarians and sponges. A mouth appeared at the perihatching embryonic stage and was renewed periodically during growth, and old mouths transformed into the body wall, thus elongating the body. Growing animals retained a small blind gut in a large body cavity without partitioning by septa and did not form tentacles, pedal discs or holdfasts externally. A growth center at the oral pole was sufficient for body patterning throughout life, and the body patterning did not show any bias from radial symmetry.

**Conclusions:**

Contrary to proposed cnidarian affinity, the *Punctatus* body plan has basic differences from that of cnidarians, especially concerning a spacious body cavity separating ectoderm from endoderm. The lack of many basic cnidarian characters in the body patterning of *Punctatus* leads us to consider its own taxonomic group, potentially outside of Cnidaria.

## Introduction

Microfossils from the earliest sediments of the Cambrian, in the Kuanchuanpu Formation, China, collectively called Small Shelly Fossils (SSFs), have provided exceptional fossils comprising reliable developmental series for some species [1], [2], [3], [4]. In particular, the species *Punctatus emeiensis* ( =  “olivooides” [3]) is outstanding in its nearly complete ontogenetic record [4], [5]. Despite the richness in number and developmental stages, however, the phylogenetic positioning of *Punctatus* is highly ambiguous because of the lack of reliable characters comparable to extant animals.

Previous reconstructions of *Punctatus* interpreted conical forms as test or thecal remains, although they were suggested to be flexible [1], [5]. As a result, an unidentified zooid was expected and the genus was allied with an extinct taxon, the conulariids [5], most of which are characterized by four-sided pyramidal skeletons bearing transverse ridges [6]. Ordovician conulariids with preserved soft tissues have polyps with Y-shaped septa in the gastric cavity and signs of strobilation [7]. General features of conulariid skeletal remains resemble periderms of modern scyphozoan coronates and the internal polyp suggests a tetra-radial pattern [6], [8]. Conulariids are thus classified as the suborder Conulariina within cnidarian Scyphozoa [8]. Tubular and pyramidal skeletal fossils coeval with *Punctatus* have also been assigned to conulariids because their transverse ribbings and longitudinal sulci between flat sides (faces) were regarded as homologous, though the pyramidal forms were six-sided [1].

Classification of *Punctatus* in previous studies relied only on the gross morphology of conical remains in spite of their rich developmental data, though recently ephyra-like fossils have been proposed to be a life stage of this animal [9]. For conical remains, their similarity to unequivocal conulariids has been tentatively proposed by referring a seemingly intermediate form [10]. Among such conulariid-like SSFs from the same sediments as of *Punctatus*, *Hexaconularia* had a preserved bivalve-like structure at the apical end. This structure was recently reinterpreted as an embryonic shell and it was suggested to be an implicated difference from conulariids [11].

Well-preserved developmental series of *Punctatus* fossils have provided detailed information of this animal both externally and internally. We have reinvestigated the developmental pattern of *Punctatus* by observing more than 10,000 fossils. Our findings show; (1) early development to gastrulation of *Punctatus* was similar to that of some cnidarians with a coeloblastula that started gastrulation as invagination or unipolar ingression [12], [13], [14], [15], (2) the oral structure developed from the blastopore, also comparable to modern ctenophores and cnidarians [16], [17], (3) the hatchling was benthic with direct development, (4) the archenteron did not line the epidermal ectoderm, but remained as a small sac-like blind gut suspended within a large blastocoel, (5) there were no gastric septa nor tentacles throughout life, (6) the mouth renewed repeatedly and was responsible for the growth of the conical body as a terminal addition, and (7) the terminal addition on the oral side resulted in the embryonic body being retained at the apical end of the conical body. These features do not match with the tubal zooid hypothesis, and instead they support that the fossils were a part of the body wall. Recent studies have revealed key features related to the development of the cnidarian body plan [16], [18], [19], [20], [21], [22], [23]. Aided by these recent studies, we reinterpret the phylogenetic position of *Punctatus*.

## Results

### Re-reconstruction of *Punctatus* Development

**Figure 1:**
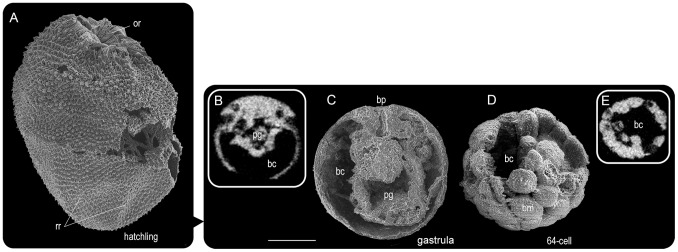
Early development of *Punctatus* deduced from hatchling features. (A) Hatchling covered with spines and developing striated oral ruffle (Sn17-18). (B) CT-section at the center of a fossil depicting a primitive gut and blastocoel (Sn40-74). (C) Split half portion of a spherical fossil with a blastopore (Sn45-46) showing proposed original structure of the primitive gut and blastocoel. (D) A well-preserved cleaving stage, possibly 64-cell stage, comparable to a typical coeloblastula with clear blastomeres (Sn25-123). (E) CT-section of specimen D showing blastocoel. Note regional differences in thickness of blastomeres. bc, blastocoel; bm, blastomere; bp, blastopore; fe, egg membrane; or, oral ruffle; pg, primitive gut; rr, radial ridge. Scale bar, 0.2 mm.

Development of *Punctatus* was reconstructed starting from the hatchling stage, as this stage displays characters linking the embryonic and post-hatching stages (Figure 1). Hatchling fossils were identified as being completely covered with stellate spines, excluding the oral region that was characterized by a striated surface [2], [3], [4], [5]. The oral pole was weakly flattened and the aboral pole tapered, and the overall shape was similar to a strawberry. Each spine was hollow and potentially was filled with cilium and/or microvilli in life [5]. Numerous spines covering the body suggest that the hatchling was benthic as spines prevent cilia-driven swimming; alternatively all cilia may have been located inside of spines [5]. This proposed benthic nature could also explain the increased chance of fossilization that we observed. The striated oral ruffle had a 10-fold radial pattern, but as two folds formed a pair, the result was a penta-radial pattern. Among many spiny globular fossils, candidates for the initial stage of oral formation were judged to be specimens completely covered with spines and radial furrows dividing the blastoporal surface into five sectors (Figure S1C), as hatchlings displayed five ridges radiating from the abblastoporal apex (Figure 1A). Most candidate specimens at earlier stages had a partially smooth surface, interpreted as the remains of an egg membrane.

Some specimens at the gastrula stage had an internal sac comparable to a primitive gut in a spacious blastocoel (Figure 1B). Subsequently, possible candidates for the gastrula and blastula stages were found (Figures 1 and S1). The specimen in Figure 1C was judged to most likely be a gastrula, suggesting an invagination type of gastrulation with some possibility of unipolar ingression, similar to extant actiniarians (sea anemones) [12], [13] and scyphozoan semaeostomes (jellyfishes with four long frilly oral arms) [14], [15]. Invagination typically occurs from the coeloblastula in extant animals, and again we found corresponding fossils. One specimen was at approximately the 64-cell stage (Figure 1D, E and Movie S1), and had almost equal cleavage with a weak gradient in blastomere size. As equal cleavage until the 64-cell stage resulting in coeloblastulae is known in modern sponges [24] and some other phyla, this specimen cannot be exclusively assigned to *Punctatus*. In cnidarians [25] and ctenophores [26], first cleavage starts from the animal pole and is easily identifiable as typically heart-shaped. However, no SSFs reliably corresponding to heart-shaped cleaving embryos have been discovered to date. The cleavage of extant ctenophores invariably produces macromeres and micromeres, and the latter cover the former during the gastrulation process [27], in contrast to the equal cleavage of *Punctatus*.

**Figure 2:**
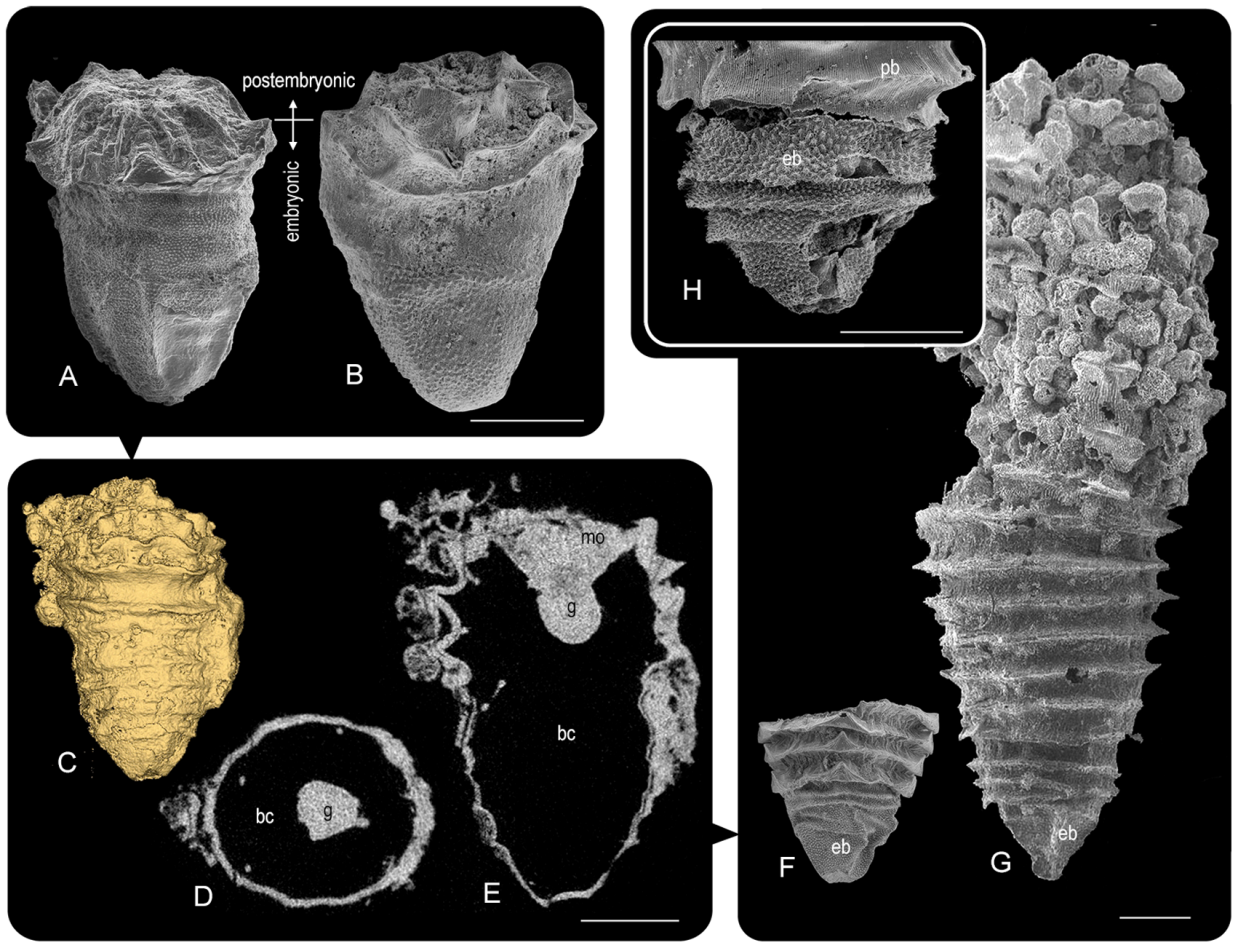
Postembryonic growth of *Punctatus*. (A) Hatchling of *P. triangulicostalis* (Sn62-11) and (B) young *P. emeiensis* with two annular fringes (kp135). Two distinct body parts are recognized in both species; the embryonic body covered with spines, and the postembryonic body with triangular processes in *P. triangulicostalis* and with striation in *P. emeiensis*. (C) Rendering from micro-CT data of a young with four annular fringes (Sn47-96), (D) transverse CT-section at gut level, and (E) median section showing a completely empty body cavity (blastocoel) and small gut. (F) Apical portion of mature *P. triangulicostalis* (Sn21-38) and (G) a mature *P. emeiensis* with more than 12 annular fringes (Sn30-03). (H) Clearly demarcated embryonic body at the tip of *P. emeiensis* (Sn27-06). bc, blastocoel (body cavity); eb, embryonic body; g, gut; mo; mouth; pb, postembryonic body. Scale bars (except C), 0.5 mm.

In postembryonic development, *Punctatus* had another characteristic feature. Surface ornamentation sharply distinguished between the two different parts of the body, one covered with spines and the other covered with fine striations. As suggested previously [4], [5], the spiny aboral pyramid was an embryonic body, and the striated region was postembryonic (Figure 2). The micro-CT analysis of a nearly intact juvenile fossil with four annual fringes revealed no structures inside the body (Figure 2C-E and Movie S2). In the body cavity, interpreted as a blastocoel based on the developmental pattern, a small and short blind gut was suspended from the mouth. Unlike cnidarians, the gut was not apposed to the epidermis and had no partitions, and unlike triploblasts there were no mesenteries extending from the body wall or parenchymal cell mass. The gut endodermal layer apparently continued into the epidermal ectoderm without any morphological disruption, suggesting that this suspended gut is not an artifactual product. These observations were confirmed with other incomplete specimens (Figure S2).

The structure interpreted in this study as the gut is also similar in appearance to the actinopharynx of actiniarians. In actiniarians, a pit on the distal end or a connecting canal to the distal cavity is expected. However, all *Punctatus* gut structures analyzed had no pits (Figures 2E and S2). Since well-grown conical fossils assignable to *Punctatus* did not show any changes in this basic pattern except for an increase in the numbers of undulations and the presence of an irregular tabula-like structure near the embryonic body in some specimens, the body plan of this juvenile specimen seems to be equivalent to that of adults. In summary, *Punctatus* eggs developed into a typical coeloblastula without micromere/macromere differentiation, and the gastrula invaginated to form a short archenteron. The gastrula body form was retained throughout life.

### Oral Formation and Growth Pattern of *Punctatus*

**Figure 3:**
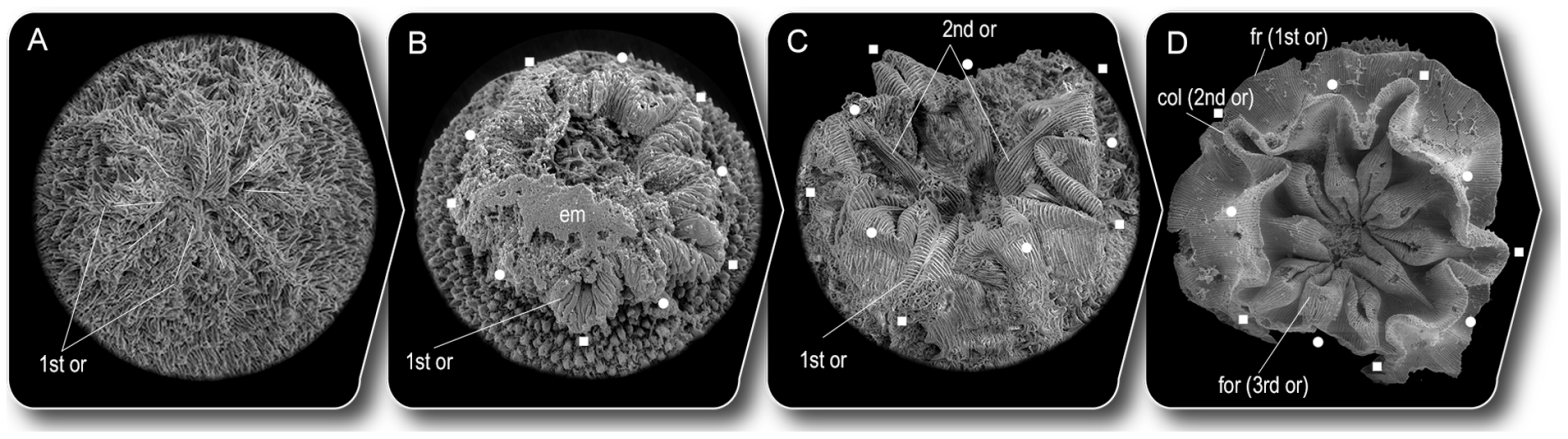
Oral development in *Punctatus emeiensis*. The mouth developed directly from blastopore. (A) Ten radial ridges as just-emerging striations surrounded with spines marginally (Sn13-109). Deca-radial pattern (white lines) conceals original penta-radial pattern. (B) Oral ruffle displaying ten petal-like folds with striation (Sn108-83) developed from stage A. The original penta-radial pattern is discernible as pairs of larger (square) and smaller (circle) folds. The specimen possibly loosened its tightly closed mouth after death. (C) New striated ruffle appears inside of the primary oral ruffle to replace it (kp08-001). (D) Functional mouth surrounded with a collar ruffle ( =  secondary oral ruffle), subsequently surrounded with a fringe of the column ( =  primary oral ruffle) (Sn24-89). The original penta-radial pattern is retained in the oral apparatus (squares and circles). col, collar; em; egg membrane; fr, fringe; for, functional oral ruffle; 1st, 2nd, 3rd or, primary, secondary and newest oral ruffles. To highlight the oral development, only the oral region is shown and scale of each image is not the same.

The mouth directly differentiated from the blastopore. The oral formation on the spiny surface started as five sectors divided by radial furrows that extended from the blastopore (Figure S1C). Between contiguous sectors there were small sectors, making 10 sectors in total. The sectored blastoporal region was gradually replaced by a striated surface proximo-distally, forming ten radial ridges (Figure 3A). The striated surface expanded further and formed an oral ruffle (Figure 3B). The striated surface of the oral ruffle was clearly distinct from the spiny embryonic surface. After hatching, the primary oral ruffle appeared to be pushed outwards and a newly forming ruffle was visible (Figure 3C). No tentacle-like structures appeared on the oral surface. Repeated ruffle formations resulted in old oral ruffles ultimately transforming into the fringed body wall via collar ruffle (Figure 3D). The fact that the oral ruffle and the surrounding collar ruffle retained the same pattern throughout life suggests that the morphogenetic ability of the oral region did not change from that originally established in development.

### Conical Remain is not a Thecal Tube, but Body Wall

Stellate surfaces first appeared as early as the blastula or early gastrula stage within the egg membrane. The fact that mouth formation took place on the stellate surface suggests that the embryonic external surface was cellular, possibly covered with a flexible cuticle that could be easily modified during development. Internal observation on a nearly intact juvenile with four annular fringes found no tabula and no zooid-like structures, consistent with other incomplete fossils (Figure 2C, E and Movie S2). In some adult conical fossils of *Punctatus*, however, a single irregular tabula-like plate was found as previously pointed out [4], but these were limited to the vicinity of the embryonic body. These observations and the ontogenetic pattern favor the interpretation that the conical form was the body wall of *Punctatus*. The tabula-like structure does not oppose our interpretation as it is possible that the aboral tip of the animal body detached from a hardened embryonic cuticle and secreted new cuticular materials to form such a tabula in grown individuals. Only the embryonic cuticle and its vicinity are theorized to have become a hard, cap-like cover.

## Discussion

The SSF family Hexangulaconulariidae, which coexisted with *Punctatus*, is a key taxon for classifying *Punctatus* as a cnidarian because it has been proposed to be a morphological intermediate between conulariids and *Punctatus* [2]. In this family, the genus *Hexaconularia* has been reinterpreted, with the apical region of the conical remains being an embryonic shell [11]. If this is the case, the persistent remaining of the embryonic body shell at the apical end of the body in *Punctatus* and *Hexaconularia* suggest that in Cambrian animals there was some developmental method that has been lost in modern, extant animals. However, this character does not necessarily imply a close affinity between these two groups. The latter did not develop radial symmetry during its embryonic development and hexa-radial symmetry appeared post-embryonically, though the lack of zooid soft tissue hampers our ability to make a conclusive judgment. Furthermore, on the theca of *Hexaconularia*, transverse ribbings that have been compared to the annular fringes of *Punctatus* were offset at the junction of the thecal faces [1], [11]. The annular fringe of *Punctatus* was, however, actually outlined as a single circle produced from the oral ruffle, suggesting different methods of formation. Although the abapical structure of Cambrian conulariids is still unknown, Ordovician forms developed plicated, triangular lappets, and lobate lappet types to close the aperture of tubes [28]. All of these structures are not inconsistent with the hypothesis of tubal zooids and their cnidarian affinity. This abapical structure in conulariids contrasts greatly with that of *Punctatus*. The oral structure of *Punctatus* developed directly from the blastopore as a soft tissue at the pre-hatching stage and was never covered with additional exoskeletal lappets.

Some extant animals produce diapause eggs or embryos ornamented with cuticle spines similar to those of *Punctatus* embryos [29], [30]. Among them, the gastrulae of freshwater hydras secrete a cuticle from blastodermal (external layer) cells. Although this shows that even early embryonic cells can secrete cuticles to form spines, the embryonic cuticle cover of hydras forms a cyst from which larvae hatch, whereas the cuticle of *Punctatus* apparently functioned as part of the body wall throughout life. Counterparts to the spiny cuticle of the embryonic body and the conical body of *Punctatus* are not found in extant cnidarians.

*Punctatus* did not have any indication of tentacles or partitioned walls inside the gastric cavity, which is also in contrast to almost all modern cnidarians. Most extant hydrozoan polypoids have no partitions, and the hydrozoan genus *Protohydra* completely lacks tentacles, with a body structure simpler than that of *Punctatus* [31], [32]. However, all solitary hydropolyps develop some structures for anchoring and the apposition of ectoderm and endoderm via mesoglea has no exception, whereas *Punctatus* did not possess such counterparts. The medusoid stage of hydrozoans displays typical tetra-radial patterning in the gastric system, as do the majority of medusozoan animals. In *Punctatus,* the morphological penta-radial pattern was prominent in the oral structure and the embryonic aboral pyramid, but the body column and the internal gut were round without conspicuous penta-radial specifications. The triangular blades arranged penta-radially in *P. triangulicostali* were repeatedly formed during the oral formation and became part of the body surface. The simple round gut in *Punctatus* contrasts with the penta-radial internal structures described in [9]. The suspended small gut detached from the body wall with no connecting structures in the body cavity favors the interpretation that *Punctatus* had no mesodermal equivalent and no matrix comparable to the mesoglea. Trabeculae occasionally observed between the epidermis and the gut show no consistent pattern(s), suggesting that they were diagenetic structures produced by bacterial activities [5].

The very simple gut of *Punctatus* raises the possibility that the animal was secondarily derived from a cnidarian ancestor with symbionts, or it was small enough to simplify its gastric cavity. It is also possible to interpret *Punctatus* as a naked animal allied with conulariids. However, among SSFs, a partitioned gastric cavity has been found in a cnidarian species smaller than *Punctatus* [33], and there are undulated conical fossils having appearances similar to *Punctatus*, but being tetra-radail with a seemingly distinct mode of development [34], [35]. The lack of tentacles in *Punctatus* may be questioned as taphonomic vias. Several well-grown individuals have preserved and intact oral ruffle soft tissues [34], [35]. Therefore, we would expect at least some specimens to have some indication of tentacles had they existed on these organisms. As these specimens have no trace of tentacles, and a polypoid fossil with filiformic tentacles has been discovered from the same sediment [3], we suggest that *Punctatus* was an animal without tentacles.

**Figure 4:**
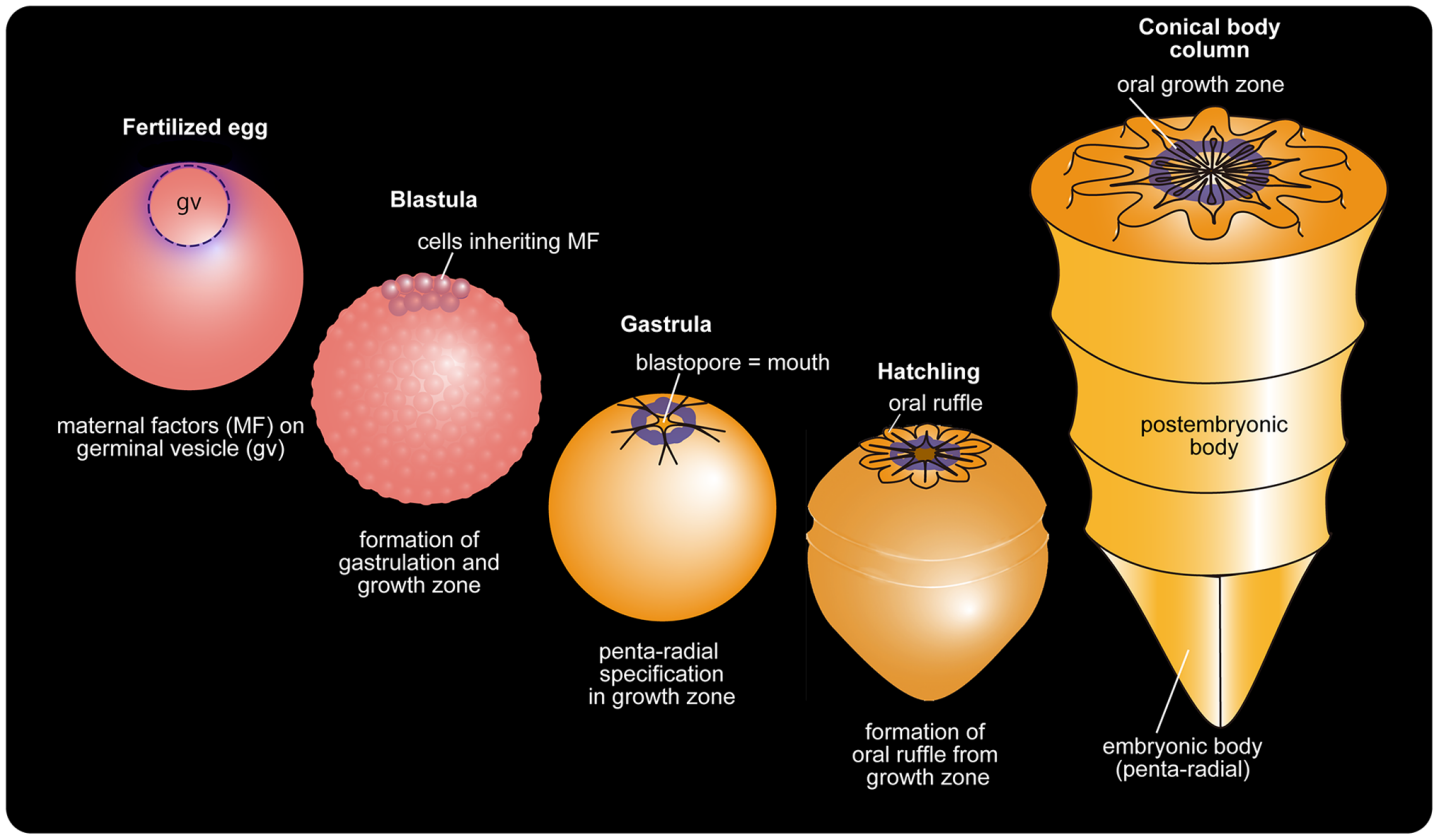
Punctatus developmental pattern based on molecular developmental data. Egg is polarized with high concentration of maternal factors at a pole, most likely the animal pole, as maternal factors tend to associate with the germinal vesicle. Depending on maternal factors, a growth zone is established at the pole where gastrulation takes place, and then the oral ruffle forms with a basic penta-radial pattern. The circular growth zone at the oral region retains its morphogenetic ability throughout life and periodically renews the oral ruffle. Old oral ruffles move to the periphery and transform into annular fringes of the column, driving the growth of the animal body. With this growth pattern, the embryonic body is retained at the tip of the body.

Judging from the spiny body surface, the *Punctatus* hatchling was immotile and benthic and thus did not require directional movement, making them free from selection pressures of body organization guided by antero-posterior polarity, unlike pelagic or epibenthic crawling cnidarian planulae, sponge larvae, and bilaterians. For *Punctatus,* a morphogenetic center at the future oral pole was sufficient to build a conical body if it could repeatedly produce oral ruffles (Figure 4). Direct development of the mouth from the blastopore is seen in extant cnidarians [16], [17] and ctenophores [36]. Once the morphogenetic center at the future oral pole in *Punctatus* was established, it may have formed oral ruffles repeatedly and elongated its conical body (Figure 4). As the gut endoderm in *Punctatus* did not line the epidermis, it is only the oral region in which the ectoderm and endoderm meet. With this body plan, unlike extant cnidarians, it would have been difficult to reproduce by strobilation. Asexual reproduction such as budding, body fission, and strobilation is common in extant cnidarians. Although fossil remains from the Kuanchunpu Formation have revealed large amounts of embryonic fossils, no trace of budding or body fission in the *Punctatus* conical body fossils has been found. This suggests that in *Punctatus* sexual reproduction was the standard mode of reproduction.

One major drawback to the present reconstruction of these fossils without tentacles and with a very small gut throughout life is a potential method of feeding. One possibility is that *Punctatus* acquired nutrients from symbionts, and such systems are often observed in modern animals. As a body plan comparable to *Punctatus* is not found in extant animals, *Punctatus* may have depended on another method of nutrition. Modern *Protohydra* lacks tentacles and has a spindle or club-shaped body when relaxed. It is slightly smaller than *Punctatus* but highly elastic and moves in a screwing motion to find prey [37]. A similar predatory behavior is unlikely for *Punctatus* as its undulated body and the lack of a holding structure does not fit with such behavior. As the sea floor in the Early Cambrian has been theorized to have been covered with a well-developed microbial mat [38], the mat may have supplied sufficient bacteria/algae that could have been caught by oral ruffle movement in microhabitats closest to the mat. A fully occupied body cavity in a minute body of *Punctatus* could function as a reservoir for nutrients, as well as a hydrostatic skeleton and hydrodynamic system to drive the oral ruffle.

### Conclusions

**Figure 5:**
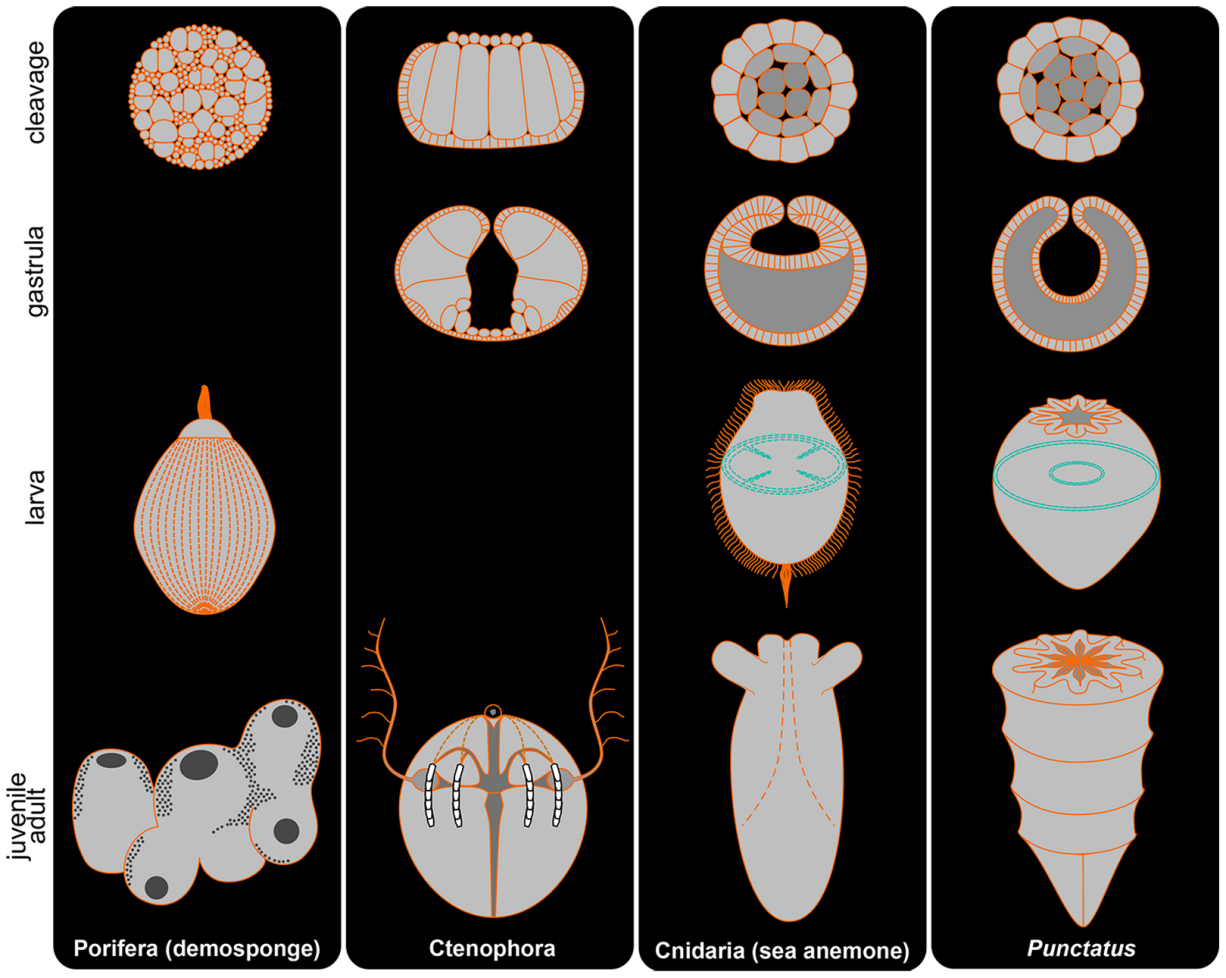
Comparison of developmental patterns among non-bilaterian phyla and *Punctatus*. The developmental pattern of *Punctatus* was similar to that of cnidarian actiniarians (sea anemones), but was generally distinct from phylum Cnidaria by the following features; no partitions of the gut, large body cavity, and benthic hatchlings. Blue broken lines in cnidarian and *Punctatus* larvae represent respective transverse sections. Porifera pattern after [41], [42] and Ctenophora pattern after [36], [43].

Among the huge variety of cnidarians, only a small number of representatives have been fully studied. Therefore it is not easy to conclude whether or not the above-mentioned characters are enough erect a new taxon separate from Cnidaria for *Punctatus*. Placozoans are very simple discoidal eumetazoans that have upper ectoderm-like and bottom endoderm-like epithelia with internal multinucleate fiber cells [39]. The two different types of the epithelia could theoretically indicate the cnidarian affinity of placozoans, even though they have no internal gut. In fact, placozoans were once regarded as secondarily simplified cnidarians [40]. Given that simple body forms make differences inconspicuous, we are in favor of the theory that *Punctatus* was a stem member of the diploblastic eumetazoans that potentially comprised an independent clade from Cnidaria due to the following reasons. Firstly, *Punctatus* grew through a unique terminal addition, retaining a gastrula-like body pattern that separated the epidermis from the small gut with a body cavity, making it distinct from Porifera, Ctenophora, and Cnidaria (Figure 5). Secondly, such development logically requires only an egg axis, and would have freed the animal from any bias of asymmetry. Finally, *Punctatus* lacks many of the basic cnidarian characters.

## Materials and Methods

All fossil specimens were collected from Kua 115–118 [3] in the Kuanchuanpu Formation of the Shizhonggou Section at Ningqiang, Shaanxi, China between 2003 and 2010. As the study area is not listed as the first or second class protection area of paleontological resources, no specific permits were required for the described field surveys (The Regulations of Protection of Paleontological Resources, Chapter Two, P. R. China). Fossil specimens with the registration numbers Sn##-### have been deposited at the Early Life Institute, Northwest University and specimens designated kp##-## at the University Museum of Geology, Chang’an University. The number of the specimens studied was greater than 10,000. The extraction of the fossils from rock samples, basic observation by SEM, and micro-CT analyses to observe internal structures were the same as those in [33]. Fossil specimens subjected to micro-CT analyses in Japan were transferred and returned to the institutions at which the fossils have been deposited by a Chinese coauthor (XY) with the permission by the Early Life Institute, Northwest University and by the University Museum of Geology, Chang’an University under the Regulations of Protection of Paleontological Resources (Chapter four).

The fossils that we relied on in the interpretation of the embryonic development were selected based on their intactness to make comparisons to equivalent developmental stages of modern animals easy to perform. This method overlooks autapomorphic features of the fossils, but can avoid the overinterpretation that is sometimes caused by metamorphoses.

## Supporting Information

Figure S1 Embryonic fossils assignable to early developmental stages of *Punctatus*. (A) Collapsing cleavage stage (Sn34-47), possibly same stage as specimen in Fig. 1D. (B) Blastula or early gastrula developing spines within the egg membrane (Sn27-06). (C) Later gastrula that has started mouth formation, showing five sectors divided by radial grooves and small sectors between grooves (Sn31-18). (D) Split half portion of a possible gastrula with blastopore, inner cell mass, and spacious blastocoel (Sn68-19). bc, blastocoel; bm, blastomere; bp, blastopore; bp (mo), blastopore (mouth); em, egg membrane; icm, ingressing or invaginating cell mass; ss, small sector. Scale bar, 0.3 mm.
(TIF)

Figure S2 Micro-CT sections showing small gut. (A) Young juvenile with a short blind gut and empty blastocoel (Sn24-88). (B) Irregular trabeculae connecting the column and short gut (Sn80-20). Trabeculae are thought to be metamorphic structures that appeared during fossilization. atr, artifactual trabecula; bc, blastocoel; col, collar; fr, fringe; g, gut; mo, mouth.
(TIF)

Movie S1 Micro-CT 3D-reconstruction of cleaving embryo. Note the wide blastocoel and the size gradient of blastomeres, suggesting an animal-vegetal axis.
(MOV)

Movie S2 Micro-CT 3D-reconstruction of young *Punctatus emeiensis* with four annular fringes. Note the very small gut suspended from the mouth and the nearly empty and spacious body cavity.
(MOV)
