# Supplementary figures and images for: A Diploblastic Radiate Animal at the Dawn of Cambrian Diversification with a Simple Body Plan: Distinct from Cnidaria?
Source: PLoS One. 2013 Jun 20;8(6):e65890. doi: 10.1371/journal.pone.0065890 (PMC3688687; doi:10.1371/journal.pone.0065890)

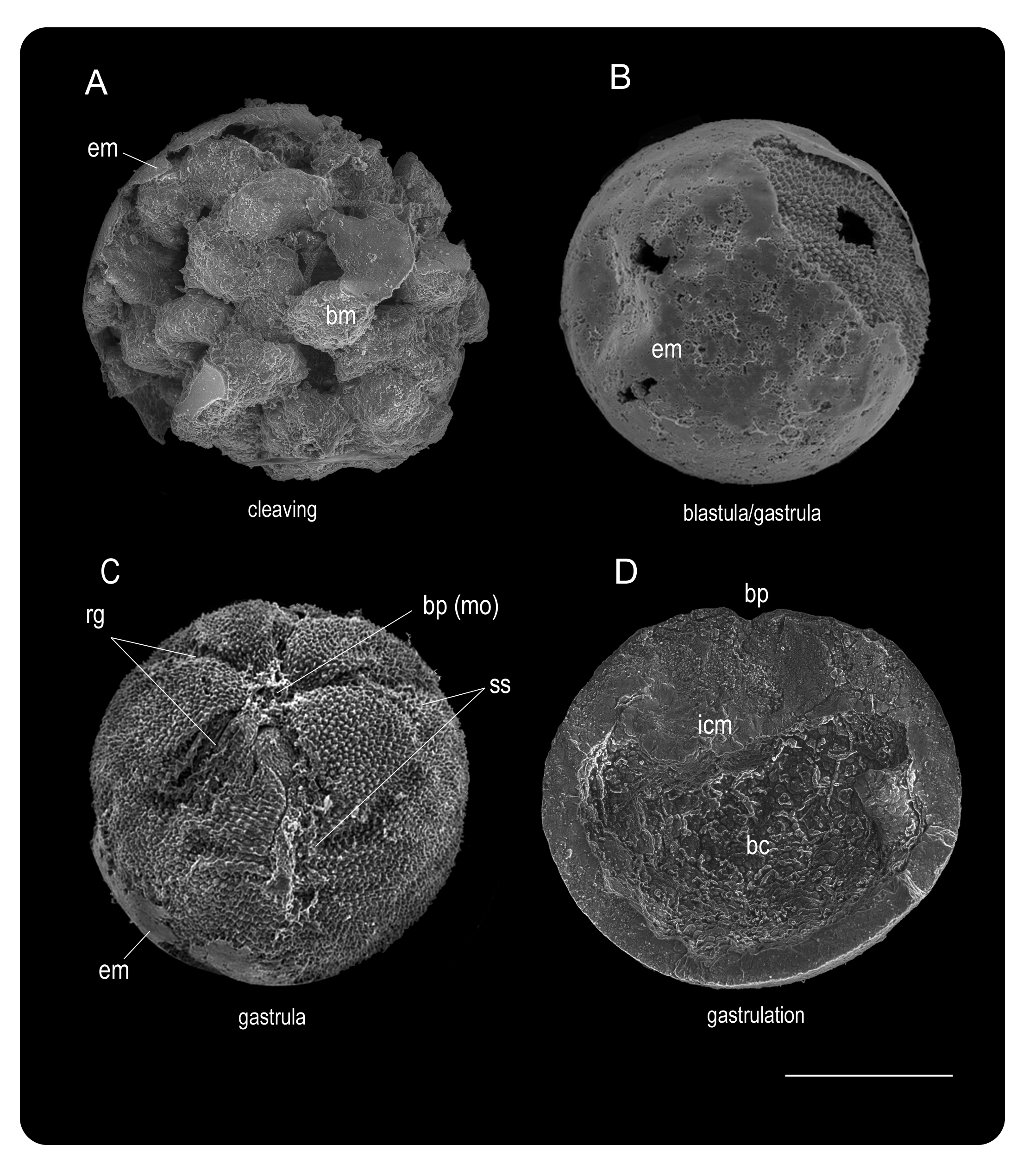

Supplement: Figure S1 — Embryonic fossils assignable to early developmental stages of Punctatus. (A) Collapsing cleavage stage (Sn34-47), possibly same stage as specimen in Fig. 1D. (B) Blastula or early gastrula developing spines within the egg membrane (Sn27-06). (C) Later gastrula that has started mouth formation, showing five sectors divided by radial grooves and small sectors between grooves (Sn31-18). (D) Split half portion of a possible gastrula with blastopore, inner cell mass, and spacious blastocoel (Sn68-19). bc, blastocoel; bm, blastomere; bp, blastopore; bp (mo), blastopore (mouth); em, egg membrane; icm, ingressing or invaginating cell mass; ss, small sector. Scale bar, 0.3 mm. (TIF) [file pone.0065890.s001.tif]

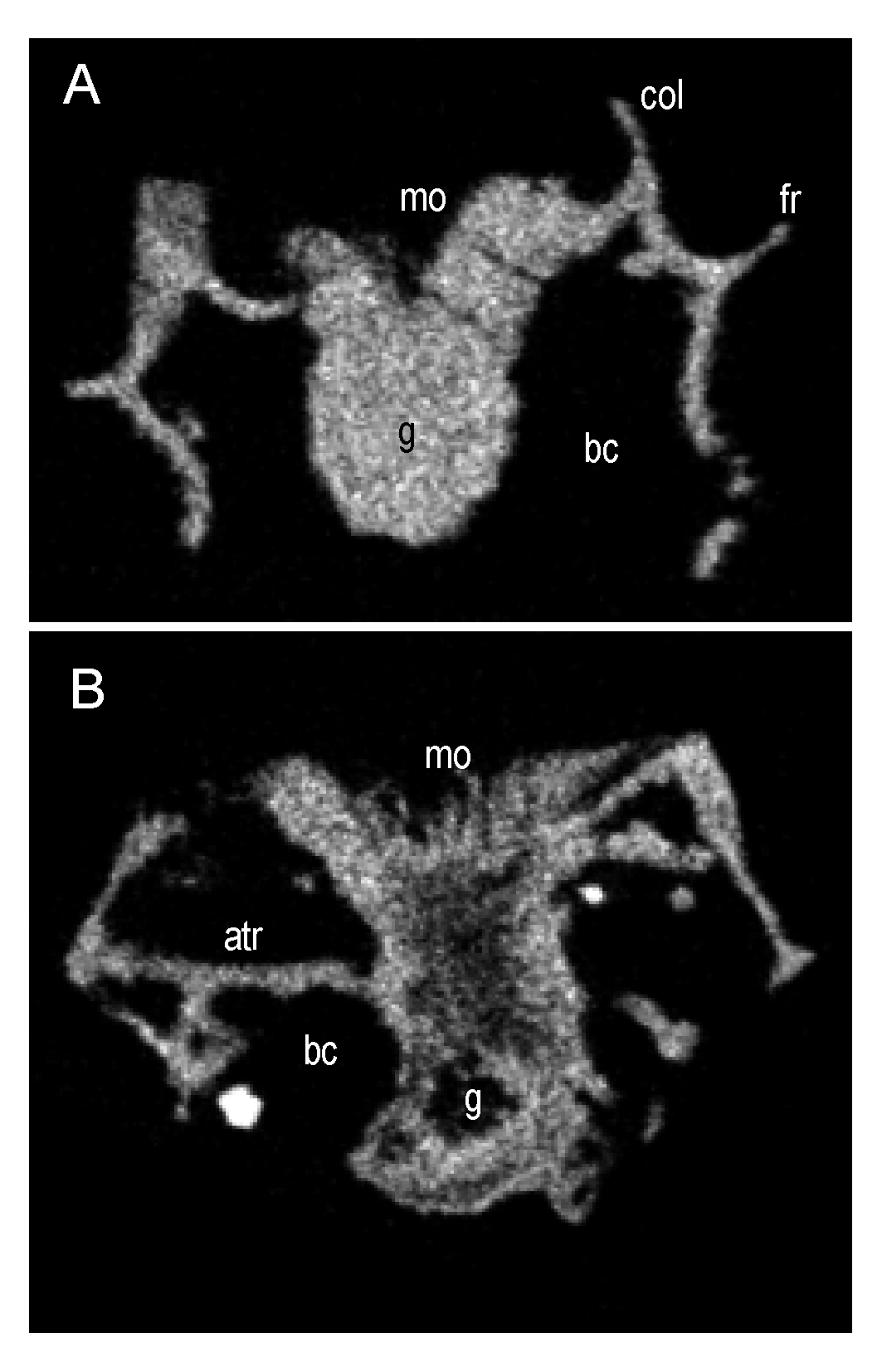

Supplement: Figure S2 — Micro-CT sections showing small gut. (A) Young juvenile with a short blind gut and empty blastocoel (Sn24-88). (B) Irregular trabeculae connecting the column and short gut (Sn80-20). Trabeculae are thought to be metamorphic structures that appeared during fossilization. atr, artifactual trabecula; bc, blastocoel; col, collar; fr, fringe; g, gut; mo, mouth. (TIF) [file pone.0065890.s002.tif]
